# A Cross-Sectional Survey in Rural Bihar, India, Indicates That Nutritional Status, Diet, and Stimulation Are Associated with Motor and Mental Development in Young Children

**DOI:** 10.3945/jn.117.251231

**Published:** 2017-06-14

**Authors:** Leila M Larson, Melissa F Young, Usha Ramakrishnan, Amy Webb Girard, Pankaj Verma, Indrajit Chaudhuri, Sridhar Srikantiah, Reynaldo Martorell

**Affiliations:** 1 Nutrition and Health Sciences Program, Laney Graduate School and; 2 Hubert Department of Global Health, Rollins School of Public Health, Emory University, Atlanta, GA; and; 3 CARE India, Bihar, India

**Keywords:** child development, mental, motor, malnutrition, nutrition, dietary diversity

## Abstract

**Background:** Many malnourished children in resource-poor settings fail to fulfill their developmental potential.

**Objective:** The objectives of this analysis were to examine the nutritional, psychosocial, environmental, and household correlates of child development in Bihar, India, and identify mediators between dietary diversity and mental development.

**Methods:** Using 2-stage cluster randomized sampling, we surveyed 4360 households with children 6–18 mo of age in the West Champaran district of Bihar. We measured motor and mental development with the use of the Developmental Milestones Checklist II. In a random subsample (*n* = 2838), we measured anthropometric characteristics and hemoglobin. Cluster-adjusted multiple linear regression analysis was used to examine the associations between nutrition indicators and development scores. Sobel’s test was used to assess significant mediators in the association between diet diversity and development scores. Analyses were stratified by children 6–11 and 12–18 mo of age.

**Results:** In all children, length-for-age *z* score (LAZ), dietary diversity, and psychosocial stimulation were significant (*P* < 0.05) correlates of motor development scores [(β coefficient ± SE) in children 6–11 mo: LAZ = 0.46 ± 0.08, dietary diversity = 0.43 ± 0.09, and stimulation = 0.15 ± 0.04; in children 12–18 mo: LAZ = 0.73 ± 0.07, dietary diversity = 0.30 ± 0.09, and stimulation = 0.31 ± 0.05] and mental development scores [(β coefficient ± SE) in children 6–11 mo: LAZ = 0.57 ± 0.10, dietary diversity = 0.84 ± 0.13, and stimulation = 0.54 ± 0.07; in children 12–18 mo: LAZ = 0.54 ± 0.11, dietary diversity = 0.40 ± 0.16, and stimulation = 0.62 ± 0.09]. Stimulation, gross motor development, and fine motor development were significant mediators in the relation between dietary diversity and mental development.

**Conclusion:** Strategies to improve dietary diversity and psychosocial stimulation could have important implications for child development of young North Indian children. This trial was registered at clinicaltrials.gov as NCT02593136.

## Introduction

Many children in low- and middle-income countries (LMICs) are exposed to malnutrition, poverty, poor health, and unstimulating environments, which detrimentally affect their development (1–3). It has been estimated that 88 million children <5 y of age in South Asia fail to fulfill their developmental potential (4); models that use UNICEF’s Early Child Development Index show that India has the largest estimated number of children 3–4 y of age (17.7 million children) with low cognitive and emotional development (3). Studies in resource-poor settings have examined the relation between nutrition and child development, many of which indicate that poor diet and nutritional status are associated with development (5, 6).

Brown and Pollitt (7) hypothesized that malnutrition affects child development through a number of mediators: child morbidity, amount of energy, motor development, and growth. Piecemeal evidence supports this model, showing that children living in healthy environments are taller, have fewer illnesses, and are more likely to explore their environment through enhanced motor skills and activity; growth and exploration are positively associated with mental development (3, 8–10). Yet the mechanisms behind these relations and their significance in Northern India remain unclear.

The Developmental Milestones Checklist-II (DMC-II) (11) is a parent report measure of infant and child motor and mental development, which has been used in parts of sub-Saharan Africa. The DMC-II is a useful tool to measure child development in resource-poor settings; it can be administered by trained nonspecialists, is simple to train on, and takes on average 15 min to complete. The objectives of the current analyses were to *1*) establish the concurrent validity of the DMC-II as a measure of child development in the context of rural Bihar, India, *2*) examine nutritional, psychosocial, environmental, and household correlates of motor and mental development, and *3*) identify mediators of the relation between diet and mental development.

## Methods

### 

#### Study setting.

Bihar, one of the poorest states in India, has a female literacy rate of only 50% (12). Child malnutrition is an important public health problem in this northern state. According to the National Family Health Survey 2015–2016, 48.3% of children are stunted, 20.8% are wasted, and 43.9% are underweight (12). The prevalence of anemia among children 6 mo to 5 y of age, although it has decreased by 14.5% points in the past 10 y, is still at 63.5% (12).

#### Study participants.

This study includes children who participated in a baseline survey for a cluster randomized trial to study the effectiveness of home fortification with multiple micronutrient powders on anemia and child development. The study was conducted in the rural West Champaran district in the state of Bihar. Four blocks (Bagaha-2, Chanpattiya, Lauriya, and Mainatand) were purposefully chosen to include 2 blocks close to and 2 blocks far from district headquarters. Within each of these 4 blocks, health subcenters (HSCs) that were prone to flooding and those prone to political difficulties were excluded. With the use of simple random sampling, a total of 70 HSCs was selected from those remaining. From September to October 2014, a baseline survey that included the DMC-II was administered in 4360 households with children 6–17.9 mo of age (half were 6–11.9 and half were 12–17.9 mo of age), which were randomly selected from a household listing. A random subsample of 2838 households were selected for additional data collection on infant anthropometry and hemoglobin (**Supplemental Figure 1**).

#### Measurements.

Child development was assessed with the use of the DMC-II (11), a 75-item parent report of gross and fine motor, language, and personal-social development. Seven questions appropriate for children 6–18 mo of age, taken from the Bayley’s Scales of Infant and Toddler Development (13) and validated (14), were added to assess cognitive development, a domain that was not available from the DMC-II. Motor development includes the sum of scores from the gross and fine motor subscales; mental development includes the sum of scores from the language, personal-social, and cognitive subscales. Items were scored based on the duration for which the child had been performing the activity at the time of the interview as follows: 1 if the child had been performing the activity consistently for the past 4 wk; 0.5 if performing the activity in the past 4 wk, but not consistently; or 0 if not yet performing the activity. This measure has been validated in Burkina Faso, Kenya, and Ghana (11, 15, 16). The Family Care Indicators, a 9-item parent report measure, previously validated in South Asia (17), was used to assess stimulating caregiving.

A Wealth Index with 5 categories was calculated with the use of principal component analysis with family assets, type of household, land ownership, and source of drinking water. A child dietary diversity score, minimum meal frequency value, and minimum acceptable diet value were created according to WHO guidelines (18). Food deprivation was assessed through mothers’ report with the use of the cross-culturally validated Household Hunger Scale (19). Households were classified as food deprived or not based on their responses to the 4-item Likert scale. Recent morbidity was measured as any fever, cough, or diarrhea in the past 2 wk reported by the caregiver. Thirty-two field investigators, trained and standardized, collected the household survey information, including the DMC-II.

Anthropometric measurements included weight, length, and midupper arm circumference (MUAC). Weight was assessed with the Seca 874 scale (Seca) and length with the Seca 417 measuring board (Seca). Length-for-age *z* scores (LAZs), weight-for-length *z* scores (WLZs), and weight-for-age *z* scores were calculated with the use of the WHO 2006 child growth standards (20); *z* scores <−2 were used to define stunting, wasting, and underweight respectively; and *z* scores <−3 were used to define severe stunting, severe wasting, and severe underweight. MUAC tapes (S0145620 MUAC, Child 11.5 Red/PAC-50) were used to measure MUAC.

Hemoglobin was measured with the HemoCue Hb 201+ Analyzar (HemoCue). Blood samples were taken by using a heel prick from children 6–11 mo of age and a finger prick for children 12–18 mo of age. Child anemia was defined as mild if hemoglobin concentration was 10 to <11 g/dL, moderate if hemoglobin concentration was 7 to <10 g/dL, and severe if hemoglobin concentration was <7 g/dL (21). If a child was found to be severely anemic, he or she was referred to the nearest primary health center for consultation.

#### Ethical considerations.

A statement about study participation, which was approved during ethical review, was read to guardians, and consent was obtained by thumb print or signature. Refusals were replaced. The study was approved by the Institutional Review Boards of the third Futures Group, Delhi, India, and Emory University and registered with the US NIH as a clinical trial (www.clinicaltrials.gov; identifier NCT02593136).

#### Quality control.

Field investigators had experience administering surveys; each had completed at least secondary schooling. Thirty-two field investigators and 10 supervisors were trained by specialists in nutrition and child development over a period of 2 wk, which included field practice. The reliability of the field investigators’ scores was established by examining the relation between the field investigators’ scores and those of an expert when assessing the same child for 5 children (20, 22). The reliability measurements for the DMC-II gave an average κ coefficient of 0.96. No single κ coefficient for any of the enumerators was <0.70. The reliability measurements for weight, height, and MUAC yielded a Pearson’s correlation coefficient between measurements of the investigators and the expert of >0.92 and a coefficient of reliability of >0.80.

The survey was translated from English into Hindi and back-translated into English. The full survey, including the DMC-II, was piloted on children from Bihar and adapted before starting the survey. The only adaptation necessary to the DMC-II was the order of questions in the personal-social subscale. During the survey, supervisors conducted 10% back-checks, which involved reinterviewing the caregiver on a random subset of questions and comparing results with the field investigators’ results, and 10% spot checks, which involved observing interviews.

#### Statistical methods.

We estimated a sample of 4340 children to have 90% power to detect a 10% difference in behavior (infant and young child feeding practices) assuming a baseline prevalence of 50%. A smaller sample size (*n* = 2800) was estimated for hemoglobin measurements to detect a 12% reduction in anemia prevalence, allowing for age stratification (children 6–11 and 12–18 mo of age). For this analysis, this sample size gave us 90% power to detect an effect size of 0.10, with the use of an α coefficient = 0.05, assuming 13 predictors in our multiple regression model.

Children with hemoglobin measurements <4 g/dL or ≥18 g/dL (0.2%) (23) and children with LAZs <−6 or >6 (0.2%), WLZs <−5 or >5 (0.6%), or weight-for-age *z* scores <−6 or >5 (0.5%) were omitted from the analysis (24).

Data were analyzed with the use of SAS version 9.4 (SAS Institute). The sample size was calculated based on the impact evaluation design. Clustering at the HSC level was accounted for with the use of PROC SURVEY statements in SAS. The use of anemia categories (none, mild, moderate, and severe) rather than continuous hemoglobin concentration did not change the results. The regression and mediation analyses were stratified by age (6–11.9 and 12–17.9 mo) because important motor and mental developmental milestones are achieved after 12 mo of age, so variance due to age could overwhelm other predictors.

Cronbach’s α coefficient for the total DMC-II score and each subscale was used to establish internal reliability, or how well questions that measured the same construct produced similar scores. Concurrent validity was examined through the sensitivity of the DMC-II to maternal education and child age with the use of Pearson’s correlation coefficients. The associations of socioeconomic, nutritional, stimulation, environmental, and household factors with motor and mental development were first evaluated with the use of univariate linear regression. A multiple regression model was then iteratively developed by retaining variables whose coefficients had *P* values < 0.05 and coefficients that did not show multicollinearity based on a test of tolerance (>0.1) and variance inflation factor (<10). Standardized coefficients (with a mean of 0 and an SD of 1) were calculated for each variable retained in the multiple regression model. Mediation analyses were performed to examine potential mediators in the relation between dietary diversity and the mental development of children 6–11 and 12–18 mo of age. Potential mediators were chosen based on Brown and Pollitt’s (7) conceptual framework of malnutrition and child development and included growth, anemia, household stimulation, and gross and fine motor development. Because recent illness was measured by parent report over the past 2 wk, it was not examined as a potential mediator in this model. Sobel’s test was used to examine significant mediation (25).

## Results

Household survey data and child development measures were collected from 4360 households. Anthropometric and hemoglobin measurements were obtained from 2838 children. Only 2 households refused the survey, and 4 children refused hemoglobin measurements.

The majority of children had a low dietary diversity score (80%), many had morbidity in the past 2 wk (76%), and 72% of children were anemic, 33% were stunted (12% were severely stunted), and 27% were wasted (7% were severely wasted) (**Table 1**). Almost all children (99%) were ever breastfed, with 95% still breastfeeding; however, only 39% of families practiced timely introduction of complementary foods at 6 mo of age, and 34% of families did not feed their children the recommended number of meals per day.

**TABLE 1:** Demographic and clinical characteristics of children 6–18 mo of age^1^

	Children 6–11 mo of age (*n* = 2208)	Children 12–18 mo of age (*n* = 2152)
Child characteristics		
Sex, F	49.7 (1096)	49.8 (1070)
Family characteristics		
Religion		
Hindu	79.1 (1745)	78.5 (1687)
Muslim	20.9 (462)	21.5 (461)
Mean maternal age, y	25.0 ± 4.8	25.4 ± 4.7
Mean paternal age, y	29.2 ± 5.5	29.7 ± 6.1
Maternal education		
Any schooling	41.2 (909)	38.5 (828)
Paternal education		
Any schooling	63.8 (1403)	64.1 (1374)
Household characteristics		
Number of preschool-aged children	2.6 ± 1.6	2.7 ± 1.6
Caste		
Scheduled caste	25.6 (564)	24.8 (533)
Scheduled tribe	8.1 (179)	8.4 (181)
Other backward caste	49.8 (1096)	51.2 (1098)
Mean Family Care Indicators score (out of 13)	4.9 ± 2.2	5.4 ± 1.0
Any food deprivation	9.8 (215)	9.1 (195)
Child nutrition indicators		
Dietary diversity	2.5 ± 1.1	3.0 ± 1.0
Score 4–7	13.4 (296)	27.4 (589)
Starches	80.5 (1777)	96.1 (2068)
Legumes and nuts	30.5 (674)	37.5 (807)
Dairy	99.7 (2202)	99.7 (2146)
Flesh foods	5.1 (112)	11.0 (237)
Eggs	2.9 (64)	4.6 (98)
Vitamin A–rich fruits and vegetables	11.2 (247)	21.7 (468)
Other fruits and vegetables	15.6 (344)	30.4 (655)
Minimum meal frequency achieved	60.8 (1315)	70.9 (1494)
Minimum acceptable diet achieved	10.8 (239)	19.7 (423)
Any recent child morbidity	76.9 (1698)	74.5 (1603)
Fever	67.1 (1482)	64.8 (1393)
Cough	56.5 (1246)	53.2 (1144)
Diarrhea	11.0 (243)	12.7 (273)
Anthropometric measurements		
Length-for-age *z* score	−1.24 ± 1.30	−1.75 ± 1.30
Weight-for-length *z* score	−1.18 ± 1.15	−1.52 ± 1.11
Weight-for-age *z* score	−1.62 ± 1.13	−1.99 ± 1.10
Midupper arm circumference	13.2 ± 1.1	13.1 ± 1.0
Anemia		
Any, hemoglobin <11 g/dL	70.5 (1002)	74.1 (1037)
Mild, hemoglobin 10 to <11 g/dL	27.7 (393)	28.3 (396)
Moderate, hemoglobin 7 to <10 g/dL	40.6 (577)	42.7 (598)
Severe, hemoglobin <7 g/dL	2.3 (32)	3.1 (43)
Child development scores		
Gross motor score (out of 22)	8.3 ± 2.8	13.1 ± 3.7
Fine motor score (out of 10)	5.7 ± 1.8	7.3 ± 1.5
Language score (out of 15)	3.1 ± 1.9	5.6 ± 2.5
Personal-social score (out of 28)	13.8 ± 3.5	17.3 ± 3.3
Cognitive score (out of 7)	3.4 ± 1.6	4.5 ± 1.4

1 Values are means ± SDs or percentages (*n*). All estimates account for cluster randomization by health subcenter. Total *n* = 4360 for all measurements (*n* = 2208 for children 6–11 mo of age and *n* = 2152 for children 12–18 mo of age) except for anthropometric measurements and anemia, for which *n* = 2838 (*n* = 1432 for children 6–11 mo of age and *n* = 1406 for children 12–18 mo of age). The minimum meal frequency was achieved if a breastfed child was fed ≥3 times or a nonbreastfed child was fed ≥4 times the previous day. Minimum acceptable diet was achieved if a breastfed child was fed ≥4 food groups and achieved the minimum meal frequency or if a nonbreastfed child received ≥2 milk feeds, was fed ≥4 food groups, and achieved the minimum meal frequency the previous day.

### Concurrent validity.

The intracluster (HSC) correlation coefficient for the DMC-II score was 0.025. Internal reliability estimates were all acceptable, with a Cronbach’s α coefficient of 0.94 for the total score, 0.92 for motor, 0.79 for language, 0.82 for personal-social, and 0.67 for cognitive subscales. Each subscale was significantly correlated with the others with the use of Pearson’s correlation coefficient. Gross motor, fine motor, language, and personal-social development scores correlated with each other, with coefficients ranging from 0.58 to 0.67. The cognitive subscale had correlation coefficients of 0.51–0.53 with gross motor, fine motor, and language scores and 0.64 with personal-social scores.

The sensitivity of the DMC-II was evaluated for children of mothers with any education compared with mothers with no education. Children of mothers with no education scored consistently lower than children of mothers with any education: mean (95% CI) score of 40.0 (95% CI: 39.4, 40.7) compared with 42.3 (95% CI: 41.6, 43.0); *P* < 0.001. Results were similar for each subscale with an effect size of 0.13 (95% CI: 0.07, 0.19) for motor, 0.20 (95% CI: 0.14, 0.26) for language, 0.18 (95% CI: 0.12, 0.24) for personal-social, and 0.27 (95% CI: 0.21, 0.33) for cognitive development.

The sensitivity of the DMC-II to age was examined by correlating scores by child age in months. Correlations were as follows: overall scores: *r* = 0.72 (*P* < 0.001), motor development score: *r* = 0.73 (*P* < 0.001); language development score: *r* = 0.5 (*P* < 0.001); personal-social development score: *r* = 0.57 (*P* < 0.001); and cognitive development score: *r* = 0.43 (*P* < 0.001).

### Child development and its correlates.

Mean gross motor scores were 10.7 (95% CI: 10.5, 10.8) out of 22; mean fine motor scores were 6.5 (95% CI: 6.4, 6.6) out of 10. The total mental development score, the sum of language, personal-social and cognitive, was 23.8 (95% CI: 23.4, 24.2) out of 50. Mean scores broken down by age group for each mental development subscale are presented in Table 1.

Among children 6–11 mo of age, multiple regression analyses indicate that motor development was significantly and positively associated with child age, LAZ, dietary diversity, WLZ, and household stimulation, but negatively associated with household food deprivation (**Table 2**). In children 12–18 mo of age, motor development was significantly and positively associated with age, LAZ, WLZ, household stimulation, and dietary diversity, but was negatively associated with female sex and caste (Table 2).

**TABLE 2:** Change in child motor development score by child condition for children 6–11 and 12–18 mo of age^1^

	Children 6–11 mo of age			Children 12–18 mo of age		
	Univariate linear regression	Multiple linear regression		Univariate linear regression	Multiple linear regression	
Age, mo	1.47*** (1.37, 1.57)	1.42*** (1.31, 1.53)	0.59	1.25*** (1.13, 1.38)	1.18*** (1.04, 1.32)	0.45
Sex, male vs. female	−0.22 (−0.48, 0.05)	—	—	−0.54** (−0.84, −0.24)	−0.72*** (−1.07, −0.37)	−0.08
Growth						
Length-for-age *z* score	0.52*** (0.37, 0.67)	0.46*** (0.30, 0.61)	0.15	0.87*** (0.70, 1.04)	0.73*** (0.59, 0.88)	0.21
Weight-for-length *z* score	0.27** (0.11, 0.43)	0.27** (0.11, 0.43)	0.08	0.66*** (0.44, 0.87)	0.50** (0.29, 0.71)	0.12
Anemia category	−0.09 (−0.30, 0.11)	—	—	0.34* (0.03, 0.64)	—	—
Other nutritional factors						
Food deprivation	−0.92*** (−1.36, −0.47)	−0.81** (−1.39, −0.22)	−0.06	−1.27*** (−1.85, −0.69)	—	—
Dietary diversity score	0.48*** (0.34, 0.63)	0.43*** (0.24, 0.61)	0.11	0.52*** (0.34, 0.70)	0.30** (0.11, 0.48)	0.07
Recent child morbidity	−0.50* (−0.89, −0.11)	—	—	−0.70** (−1.12, 0.28)	—	—
Household stimulation score	0.25*** (0.18, 0.33)	0.15** (0.07, 0.23)	0.08	0.38*** (0.28, 0.49)	0.31*** (0.20, 0.42)	0.16
Distal factors						
Wealth index quintile	0.21** (0.10, 0.32)	—	—	0.45*** (0.31, 0.59)	—	—
Any maternal education	0.64** (0.33, 0.95)	—	—	1.10*** (0.79, 1.41)	—	—
Any paternal education	0.52*** (1.37, 1.56)	—	—	0.95*** (0.58, 1.32)	—	—
Caste	0.09 (−0.09, 0.26)	—	—	−0.23* (−0.45, −0.02)	−0.26** (−0.44, −0.09)	−0.07

1 Values are coefficients (95% CIs). All estimates account for cluster randomization by health subcenter and are adjusted for age of child. Anemia was categorized as moderate or severe (hemoglobin <10 g/dL), mild (hemoglobin 10 to <11 g/dL), and no anemia (hemoglobin ≥11 g/dL). Recent child morbidity includes fever, cough, or diarrhea in the past 2 wk. Multiple regression model iteratively developed with variables retained if the *P* value for their coefficient remained <0.05. Regression coefficient standardized with a mean of 0 and an SD of 1. *R*^2^ = 0.42 for the multiple regression model in children 6–11 mo of age with motor development as the outcome. *R*^2^ = 0.33 for the multiple regression model in children 12–18 mo of age with motor development as the outcome. *P < 0.05, **P < 0.01, ***P < 0.001.

Multiple regression analyses in the younger group (children 6–11 mo of age) showed that mental development scores were positively and significantly associated with LAZ, dietary diversity, household stimulation, and maternal education, but were negatively associated with being food deprived (**Table 3**). In the older group (children 12–18 mo of age), mental development was similarly associated with age, LAZ, household stimulation, dietary diversity, household food deprivation, maternal education, and WLZ (Table 3).

**TABLE 3:** Change in child mental development score by child condition for children 6–11 and 12–18 mo of age^1^

	Children 6–11 mo of age			Children 12–18 mo of age		
	Univariate linear regression	Multiple linear regression		Univariate linear regression	Multiple linear regression	
Age, mo	1.81*** (1.66, 1.97)	1.59*** (1.42, 1.76)	0.45	1.35*** (1.19, 1.51)	1.19*** (1.00, 1.37)	0.34
Sex, male vs. female	−0.36 (−0.87, 0.15)	—	—	−0.19 (−0.51, 0.13)	—	—
Growth						
Length-for-age *z* score	0.80*** (0.61, 1.00)	0.57*** (0.37, 0.76)	0.12	0.82*** (0.59, 1.05)	0.54*** (0.32, 0.75)	0.12
Weight-for-length *z* score	0.30* (0.06, 0.53)	—	—	0.61*** (0.33, 0.89)	0.35** (0.11, 0.60)	0.07
Anemia category	−0.46 (−1.07, 0.15)	—	—	0.57 (−0.20, 1.33)	—	—
Other nutritional factors						
Food deprivation	−1.56*** (−2.29, −0.83)	−1.48** (−2.40, −0.56)	−0.07	−2.41*** (−3.38, −1.44)	−1.43** (−2.42, −0.44)	−0.07
Dietary diversity score	0.96*** (0.73, 1.19)	0.84*** (0.58, 1.10)	0.15	0.77*** (0.49, 1.06)	0.40* (0.07, 0.72)	0.07
Recent child morbidity	−0.03 (−0.59, 0.54)	—	—	−0.99** (−1.67, −0.31)	—	—
Household stimulation score	0.71*** (0.58, 0.83)	0.54*** (0.39, 0.69)	0.20	0.70*** (0.55, 0.85)	0.62*** (0.44, 0.81)	0.24
Distal factors						
Wealth index quintile	0.62*** (0.44, 0.81)	—	—	0.72*** (0.50, 0.94)	—	—
Any maternal education	1.86*** (1.45, 2.27)	0.97** (0.40, 1.54)	0.08	1.61*** (1.15, 2.06)	0.64* (0.01, 1.27)	0.05
Any paternal education	1.27*** (0.83, 1.72)	—	—	1.32*** (0.78, 1.85)	—	—
Caste	0.18 (−0.07, 0.43)	—	—	−0.19 (−0.52, 0.14)	—	—

1 Values are coefficients (95% CIs). All estimates account for cluster randomization by health subcenter and are adjusted for age of child. Anemia is categorized as moderate or severe (hemoglobin <10 g/dL), mild (hemoglobin 10 to <11 g/dL), and no anemia (hemoglobin ≥11 g/dL). Recent child morbidity includes fever, cough, or diarrhea in the past 2 wk. Multiple regression model is iteratively developed with variables retained if the *P* value for their coefficient remained <0.05. Regression coefficient is standardized with a mean of 0 and an SD of 1. *R*^2^ = 0.36 for the multiple regression model in children 6–11 mo of age with mental development as the outcome. *R*^2^ = 0.25 for the multiple regression model in children 12–18 mo of age with mental development as the outcome. *P < 0.05, **P < 0.01, ***P < 0.001.

### Mediation between dietary diversity and mental development.

**FIGURE 1:**
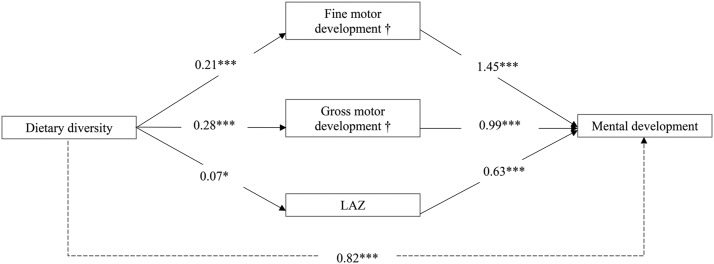
Mediation analysis between dietary diversity and mental development in children 6–11 mo of age. Values are unstandardized coefficients. Covariates include age, household wealth, maternal education, paternal education, recent morbidity, caste, and food deprivation. All models account for cluster randomization by health subcenter. Weight-for-length *z* score, household stimulation, and anemia were not examined as potential mediators in children 6–11 mo of age because they were not significantly associated with dietary diversity or the outcome. Solid arrows represent the associations for each mediator separately. Associations between dietary diversity and mediators are adjusted for covariates, and associations between mediators and mental development are adjusted for dietary diversity and covariates. Dashed arrow represents association adjusting for covariates. *P < 0.05, ***P < 0.001. ^†^Significant mediator with the use of Sobel’s test. LAZ, length-for-age *z* score,.

**FIGURE 2:**
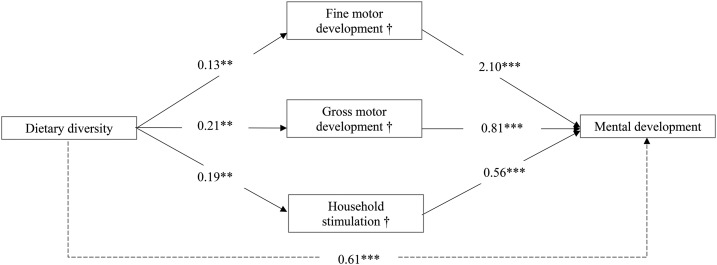
Mediation analysis between dietary diversity and mental development in children 12–18 mo of age. Values are unstandardized coefficients. Covariates include age, household wealth, maternal education, paternal education, recent morbidity, caste, and food deprivation. All models account for cluster randomization by health subcenter. Weight-for-length *z* score, length-for-age *z* score, and anemia were not examined as potential mediators in children 12–18 mo of age because they were not significantly associated with dietary diversity or the outcome. Solid arrows represent associations for each mediator separately. Associations between dietary diversity and mediators are adjusted for covariates; and associations between mediators and mental development are adjusted for dietary diversity and covariates. Dashed arrow represents association adjusting for covariates. **P < 0.01, ***P < 0.001. ^†^Significant mediator with the use of Sobel’s test.

Mediation analyses demonstrated that, in both age groups, gross motor and fine motor development were significant mediators of the relation between dietary diversity and mental development; household stimulation was a significant mediator in the older age group only (**Figures 1** and **2**, **Supplemental Table 1**). The results show a direct association between dietary diversity and mental development, with every addition of 1 diet group associated with a mean increase of 0.82 and 0.61 points in mental development scores for children 6–11 and 12–18 mo of age, respectively. The coefficients for the indirect associations between dietary diversity and mental development through gross motor, fine motor, and household stimulation are displayed in Figures 1 and  2. Similar results were obtained when mental development was separated into language and personal-social and cognitive development.

## Discussion

Our contributions to knowledge about nutrition and child development in LMICs include the following: *1*) establishing the sensitivity of the DMC-II to child age and maternal education in the context of Northern India in children 6–18 mo of age; *2*) determining that LAZ, dietary diversity, and household stimulation are important correlates of motor and mental development; and *3*) identifying household stimulation and gross and fine motor development as significant mediators in the relation between dietary diversity and mental development.

The validity of the DMC-II in the context of rural Northern India was established by its sensitivity to maternal education and child age. The effect sizes for motor and personal-social subscale scores by maternal education were small but significant and replicate previous findings in Burkina Faso (11). The cognitive subscale that we added has also been examined in Ghana with similar internal and external validity results (14). Cognitive scores were strongly correlated with personal-social developments scores (measuring reaction to others, recognition of others, play, dressing, eating and drinking, and toilet training), suggesting that they measure abilities that develop concurrently and, in addition to language, contribute to a coherent score of mental development.

We hypothesized that psychosocial stimulation, growth, and nutritional factors were important correlates of child development. We found that household stimulation is a strong correlate of mental development, but is less so for motor development, which is consistent with other literature showing that a responsive and stimulating environment creates engaging situations for children to develop their cognitive, language, and social abilities (5, 26). The LAZ was a more important correlate for development than WLZ. A low LAZ has repeatedly been associated with reduced motor and mental skills (4, 27), such that it is often used as a proxy of child development (4). A meta-analysis of data from 29 LMICs also determined that the LAZ was significantly associated with cognitive abilities, age of walking, and motor skills (28). Our results show that dietary diversity is a more important correlate of development than food deprivation, which defines severe household nutritional stress. After adjusting for other significant correlates, food deprivation remains independently associated with development, and ours is the first study, to our knowledge, to document this in an LMIC setting. Although food deprivation may indirectly affect motor and mental development through undernutrition and illness (29), it may also be detrimental to maternal mental health (30, 31) and affect the amount of stimulation, care, and nutrition received by a child (32). Recent morbidity, anemia, and distal factors, such as household wealth and paternal education, were correlates of development when controlling for child age only, but were no longer significant in the adjusted models. This finding could be due to their strong correlation with LAZs, dietary factors, and household stimulation (33–37).

The specific mechanism responsible for the relation between diet and development was explored through mediation analysis. Low dietary diversity was common and is potentially modifiable through community-based nutrition programs, which would enhance nutrition of children. The diet of young children in rural Bihar is monotonous, and greater diversity in foods eaten could improve overall nutrition and development. The mediation analysis indicates that stimulation in children 12–18 mo of age as well as fine and gross motor development in children 6–18 mo of age are mediators in the relation between dietary diversity and mental development. Dietary diversity could influence the amount of stimulation in the home through several mechanisms: first, a child’s diet is highly correlated with that of their mother (38), who is often the primary source of stimulation; and second, a mother who supplies a diverse diet to her child likely also supplies diverse stimulation (39). Diet and stimulation are more diverse in children 12–18 mo of age, and this may explain why stimulation is a significant mediator only in this older group. At this age, when a child starts walking and becoming increasingly active, the mother may become more aware of their child’s stimulation needs and make materials more available. The significant mediation through motor skills demonstrated in this analysis may occur in a number of ways. Dietary diversity could build muscle through increased nutrients, including iron and animal-source foods, which in turn could contribute to improved physical activity, initiative, and leadership and social behaviors (40, 41). The resulting motor skills allow children to provide their own stimulation, leading to the development of mental abilities (42). A more active child is also more likely to receive attention, be spoken to, and be heard by parents and others (39). In addition, many personal-social and cognitive skills require fine motor coordination (e.g., putting on clothes or rearranging small objects). Lastly, a child with more advanced motor skills may appear more mature and may be given more sophisticated stimulation (7). Previous work shows that nutrition can increase motor abilities and thereby children’s interaction and exploration of their environment (7–9, 43), thus enhancing their mental development, particularly when their experiences are mentally challenging (8). This is one of the first studies, to our knowledge, to show that all 3 aspects are connected.

The mediation analysis in this article builds on previous work that established a framework linking nutrition and development (7, 43). However, the directionality of these relations are hypothetical, and the cross-sectional nature of this study does not allow for inferences of causality. For instance, greater fine motor skills could lead to more self-feeding, making the child appear more mature and ready for a more diverse diet.

Limitations of this analysis include the use of a parent-report measure of child development. Some bias could be introduced if mothers who recall certain motor skills are better at recalling mental abilities, or if mothers who are more involved in the development of their child are better able to recall activities and abilities. A more detailed examination of morbidity will be important in examining the mechanisms linking diet and child development in future studies, for instance, through an account over a longer period and obtaining data on total number of days sick and severity of the illness. Lastly, the survey was performed in September and October, which are harvest months in Bihar. If the survey were performed during a less plentiful time, relations between development and WLZ or morbidity may be different.

Despite these limitations, this analysis reveals important correlates of motor and mental development in young children. With the caveat that the study areas excluded those prone to flooding and political difficulties and therefore exclude areas that are potentially economically worse off, these findings could be generalizable to other parts of rural India with similar nutritional and economic indicators to Bihar. State programs in India that work on improving nutrition in early life, such as the National Nutrition Mission, could affect child development by focusing on diversification of complementary foods. The mediation models extend the current framework for nutrition and child development (7, 43) by identifying both fine and gross motor abilities as significant mediators in the relation between diet and mental development and by identifying other nutritional indicators that drive the development of mental abilities in children 6–18 mo of age. Additional research examining a path analysis of the full theoretical model may help elucidate the indirect as well as direct pathways between nutrition and mental development.

Malnutrition is an important problem in LMICs with detrimental effects on child development. Our findings suggest that nutrition programs that target diet diversification can have important implications for the mental development of young Indian children through their benefits on household stimulation and motor skills.
